# Laparoscopic Nephroureterectomy: Oncologic Outcomes and Management of Distal Ureter; Review of the Literature

**DOI:** 10.1155/2009/826725

**Published:** 2008-11-05

**Authors:** Andre Berger, Amr Fergany

**Affiliations:** Section of Laparoscopic and Robotic Surgery, Cleveland Clinic Foundation, Glickman Urological Institute, Cleveland, OH 44195, USA

## Abstract

*Introduction*. Laparoscopic radical nephroureterectomy (LNU) is being increasingly performed at several centers across the world. We review oncologic outcomes after LNU procedure and the techniques for the management of distal ureter. *Materials and Methods*. A comprehensive review of the literature was performed on the oncological outcomes and management of distal ureter associated with LNU for upper tract transitional cell carcinoma (TCC). *Results and Discussion*. LNU for upper tract TCC is performed pure laparoscopically (LNU) or hand-assisted (HALNU). The management of the distal ureter is still debated. LNU appears to have superior perioperative outcomes when compared to open surgery. Intermediate term oncologic outcomes after LNU are comparable to open nephroureterectomy (ONU). *Conclusions*. Excision of the distal ureter and bladder cuff during nephroureterectomy remains controversial. Intermediate term oncologic outcomes for LNU compare well with ONU. Initial long-term oncologic outcomes are encouraging. Prospective randomized comparison between LNU and open surgery is needed to define the role of these modalities in the current context.

## 1. Introduction

Upper tract TCC accounts for 5% of all urothelial tumors [1]. It usually occurs in patients older than 60. Compared to bladder cancer, upper tract TCC is diagnosed more frequently at advanced stages. In most series, in almost half the patients, tumor stages at diagnosis have been described as pT2 or higher. Stage is the main predictor for survival. Delay in diagnosis and treatment is related to worse prognosis. Standard management consists of open radical nephroureterectomy (ONU), which usually requires one large or two separate abdominal incisions. Since LNU was first reported by the Washington University Group in 1991 [2], the benefits of this procedure regarding perioperative morbidity, cosmesis, and convalescence have been established [3–5]. Mainly, there are 2 laparoscopic approaches: pure laparoscopic nephroureterectomy (LNU) and hand-assisted nephroureterectomy (HALNU). Despite the perioperative advantages, some oncological issues remain unclear, mainly management of the distal ureter and the role of lymphadenectomy.

## 2. Materials and Methods

We performed an extensive National Library of Medicine database search with no date restriction using the keywords upper tract transitional cell carcinoma, nephroureterectomy, and laparoscopic nephroureterectomy. Of the over 100 papers identified, 25 of each were selected for this review on the basis of their contribution in advancing the field with regards to (1) evolution of concepts (2) development and refinement of techniques, and (3) intermediate oncological outcomes.

## 3. Results and Discussion

### 3.1. Management of Distal Ureter

The management of the distal ureter is still controversial. The open extravesical or transvesical approach is accepted as the most oncologically safe. However, patient factors as obesity and previous history of pelvic surgery or radiotherapy can make ureteral excision more difficult. In 1952, McDonald et al. first reported an endoscopic method to handle the distal ureter [6]. In the laparoscopic era, many attempts have been made to avoid the open approach to the distal ureter, which is still commonly used. Shalhav et al. [4] described the laparoscopic stapling of the distal ureter and bladder cuff and positive margins were associated with the method. Matin et al. compared the outcomes (median follow-up 23 months) using the two different techniques of en bloc excision of bladder cuff: 36 patients underwent technique of cystoscopic intravesical secured detachment of en bloc bladder cuff and juxtavesical ureter using needlescopic instruments percutaneouslly [7] and 12 underwent laparoscopic extravesical stapling. The stapling technique was associated with a decreased overall survival, decreased recurrence free survival, and higher positive surgical margin rate. Kurzer et al. (2006) evaluated 49 patients on a mean follow-up of 10.6 months and reported their results after cystoscopic circumferential excision of the distal ureter without primary closure of the bladder cuff with simultaneous ureteral ligation during HALNU [8]. No cases of local pelvic or peritoneal recurrences were reported. Vardi et al. (2006) reported a new technique to manage the distal ureter [9]. They proposed an en bloc excision of the bladder cuff and juxtavesical ureter during HALNU using a flexible cystoscope and a 5F electrode without repositioning the patient. Mean follow-up was 31 months (range 5–44) and none of the 6 patients presented with local recurrence. Recently, Nanigan et al. (2006) reported using robotic assistance in an attempt to decrease the technical challenge of excision of distal ureter in 11 patients [10]. As part of the procedure, they filled the bladder with a saline solution before opening it and aspirated all the fluid to avoid dissemination of cancer cells. In addition to the disadvantage of increased cost, the 6-month follow-up is not enough to evaluate local recurrence. Agarwall et al. (2008) modified the Cleveland Clinic technique. They performed a circumscribed incision in the ureteric orifice with a bladder cuff using a Collins knife. The ureter stump was ligated with an endoloop via cystoscope to avoid urine leak from the upper tract. Complete excision was achieved in all 13 patients. Five patients had bladder recurrence, 2 close to the ureteral scar [11].

Since most studies do not show any difference between different methods of handling the distal ureter, the best option is to follow individual surgeon's preference as long as the fundamental oncological concepts are preserved: having a complete resection of the distal ureter with bladder cuff and avoiding tumor spillage.

### 3.2. Oncological Outcomes after LNU

Laparoscopic nephroureterectomy is performed utilizing the same surgical principles as laparoscopic radical nephrectomy. A transperitoneal or retroperitoneal approach can be chosen. Most surgeons are familiar with the transperitoneal approach, which has the advantage of allowing dissection of the ureter all the way to the bladder. This is essential if endoscopic management of the distal ureter (as previously described) is planned. Surgeons familiar with the retroperitoneal approach to radical nephrectomy can perform the renal part of the operation retroperitoneally, although access to the distal ureter is difficult with this approach. This is best suited to cases where the distal ureter will be managed through an open approach. In either case the ureter is not divided and left in continuity. A clip placed on the ureter will minimize the risk of tumor seeding resulting from manipulation of the kidney. In cases of ureteric tumors, careful attention to wide dissection of the ureter is essential to avoid a positive margin or entry into the ureter with tumor spillage.

Long-term follow-up after ONU is well documented in some large series. Charbit et al. (1991) reported the first big follow-up series with upper tract TCC in 108 patients [17]. Survival rates after 5 and 10 years were 67% and 65%, respectively. Hall et al. (1998) reviewed 252 patients after ONU (median follow-up 64 months). Recurrence occurred in 67 (27%) patients and urothelial recurrences represented 69% of total [18]. Median time to recurrence was 12 months. Actuarial 5-year cancer-specific survival rates by primary tumor stage were 100% for Ta/Cis, 92% for T1, 73% for T2, and 41% for T3. Median survival for pT4 patients was 6 months. On multivariate analysis, tumor stage was a significant predictor for recurrence, whereas patient age and stage were significant predictors for survival. In contrast, a multicenter study by Ozsahin et al. (1999) evaluated 126 patients with upper tract TCC (median follow-up 39 months) and reported poor oncological outcomes of ONU [19]. In a median period of 9 months, 66% of the patients recurred. The 5- and 10-year overall survivals were 29% and 19%, respectively. Multivariate analysis revealed that independent prognostic factors influencing outcome were T staging, positive surgical margin, and tumor in the ureter. Lower survival rates in this study may be explained by high proportions of high grade (76%), nonorgan confined disease (59%), and positive surgical margin (26%).

Long-term follow-up studies after LNU are still sparse. El Fettouh et al. (2002) reported the results of 116 patients who underwent LNU on a multicenter basis with a median follow-up of 25 months [12]. Positive margins were identified in 4.5% of patients, local recurrence in 1.7%, bladder recurrence in 24%, and mean time to recurrence was 13.9 months. Distant metastasis rate was 9%; mean time to metastasis was 13 months. Two-year cancer-specific survival was 87%. According to T stage, 2-year cancer specific survival was 89% for pT1, 86% for pT2, 77% for pT3, and 0% for pT4. Muntener et al. (2007) reported the outcomes of 39 patients after LNU (median follow-up 74 months). Five-year cancer specific survival was 68%. Tumor stage was the only factor related to cancer death and ureteral tumor was the only factor associated with recurrence [13].

Cohorts comparing perioperative and short/intermediate oncological outcomes between ONU and LNU have been published. Bariol et al. (2004) evaluated 25 patients who underwent LNU and 42 who underwent ONU for TCC in a median follow-up of 101 and 96 months, respectively [14]. Local and bladder recurrence rates were 28% (7 patients) for LNU and 42% (15 patients) for ONU, while more ureteral tumors were described in ONU. One and 5-year metastases-free survivals were 80% and 72% for LNU and 87% and 82% for ONU, while no statistical difference between the two surgical treatments was found. Rouprêt et al. (2007) compared 20 patients who underwent LNU (median follow-up 68.5 months) to 26 who underwent ONU (median follow-up 78 months). Recurrence occurred in 20% of cases of LNU and 53% of ONU [15]. Median time to recurrence was 15 and 18 months, respectively. Five-year cancer-specific survival was 90% and 61% and 5-year recurrence-free survival was 71% and 51%, respectively. Okegawa et al. (2006) compared 25 LNU (mean follow-up 24 months) and 23 ONU (mean follow-up 29 months). In LNU and ONU groups, recurrence rates were 20% and 17% and mean time to recurrence were 9.5 and 23.4 months [20]. Distant metastasis rate was 8% for LNUX and 13% for ONUX. Two-year cancer-specific survival was 91% for LNU and 89% for ONU. No significant difference was detected in recurrence-free survival and cancer-specific survival. Manabe et al. (2007) evaluated 58 patients after LNU (mean follow-up 13.6 months) and 166 after ONU (mean follow-up 28 months). Bladder recurrence was reported in 33% of patients after LNU and in 38% after ONU [16]. Distant metastases were reported in 17% and 20% of the patients, respectively. The 2-year recurrence-free survivals were 76% and 82%. No difference was found in cancer-specific survival.

Some recent series report results after hand-assisted LNU (HALNU), but oncologic outcomes are limited. Wolf et al. evaluated 54 patients who underwent HALNU with median follow-up of 25 months [21]. Urothelial recurrences occurred in 66% patients. History of bladder tumors was associated with urothelial recurrence. Nonurothelial recurrences were found in 25% of the patients at a mean 10.4 months follow-up. Age and grade correlated with nonurothelial recurrence. The 2 and 3-year cancer-specific survival were 86% and 80%, respectively. Organ-confined disease and nonorgan-confined disease were associated with a 3-year survival of 100% and 36%, respectively. High-grade disease correlated with poorer 3-year cancer-specific survival. Chung et al. (2007) also described comparable recurrence free, 3-year cancer-specific and overall survivals in 39 patients after HALNU (median follow-up 48 months) and 41 patients after ONU (median follow-up 62 months) [22].

The propensity for dissemination of high-grade TCC is well known. An important concern with laparoscopic approach is port site metastasis. Seven cases have been published so far. No surgical bag was used in six cases and the surgical bag was torn during retrieval of the specimen in one case [22].

Recently, the importance of extended lymph node dissection for bladder cancer regarding staging and prognosis has been established. Given the histological similarity between bladder cancer and upper tract TCC, lymphadenectomy should also be important for the management of upper tract TCC. Kondo et al. (2007) evaluated 169 patients that underwent open nephroureterectomy divided in 3 different groups: complete lymphadenectomy, incomplete lymphadenectomy, and no lymphadenectomy [23]. Extended lymphadenectomy improved survival in patients with pT3 stage or higher. On multivariate analysis, complete lymphadenectomy, T stage and grade were significant prognostic factors for cancer-specific survival. Brausi et al. (2007) reported retroperitoneal lymph node dissection and T stage as the only independent significant prognostic factors on overall survival [24]. Busby et al. (2006) found no difference between ONU and LNU concerning number of lymph nodes retrieved, median number of positive nodes retrieved, and median density of positive nodes, showing that lymphadenectomy can be performed in the laparoscopic approach as adequately as in open approach [25].

Intermediate oncological outcomes after LNU are similar to ONU. Series combining long follow-up and large number of patients are lacking. Finally, to confirm these previous encouraging findings, prospective randomized trials are still needed Table 1 summarizes the oncological outcomes after LNU.

## 4. Conclusion

Many centers worldwide are now performing LNU. Contemporary series have demonstrated technical feasibility and safety. The management of distal ureter is still debated. Although large series of 5-year oncologic data are not yet available in the LNU literature, reports indicate that intermediate and long-term oncological outcomes are similar to ORC. Carefully designed prospective randomized trials comparing LNU and ONU are necessary to define the role of these modalities in the current and future management of upper tract TCC.

## Figures and Tables

**Table 1:** LNU series and comparative studies.

	Patients (n) LNU/ONU	Median follow-up (mo)	Recurrence (%)	Local recurrence (%)	Bladder recurrence (%)	Distant metastasis (%)	Overall survival (%)	Cancer-specific survival (%)	Risk factor associated to survival (%)
*LNU X Series*									

El Fettouh (2002) [12]	116	25	Not stated	Not stated	24	9	Not stated	87 (2 year)	Not stated
Muntner (2007) [13]	39	74	46	5	Not stated	18	59 (5 year)	68 (5 year)	Tumor stage

*LNU versus open surgery*									

Bariol (2004) [14]	58 26/22	101/96	Not stated	8/15	28/ 42	28/18	56/59	72/82 (5 year)	Not stated
Rouprêt (2006) [15]	46 20/ 26	68/ 78	19 (urothelial)	Not stated	Not stated	10/35	Not stated	90/61(5 year)	Tumor stage and grade
Manabe (2007) [16]	224 58/166	14/ 28 (mean)	33/38	1 case port/ 2 cases	Not stated	17/20	84/84 (2 year)	85/82 (2 year)	Tumor stage and grade

## References

[1] Tawfiek ER, Bagley DH (1997). Upper-tract transitional cell carcinoma. *Urology*.

[2] Clayman RV, Kavoussi LR, Figenshau RS, Chandhoke PS, Albala DM (1991). Laparoscopic nephroureterectomy: initial clinical case report. *Journal of Laparoendoscopic Surgery*.

[3] Gill IS, Sung GT, Hobart MG (2000). Laparoscopic radical nephroureterectomy for upper tract transitional cell carcinoma: the Cleveland clinic experience. *The Journal of Urology*.

[4] Shalhav AL, Dunn MD, Portis AJ, Elbahnasy AM, McDougall EM, Clayman RV (2000). Laparoscopic nephroureterectomy for upper tract transitional cell cancer: the Washington University experience. *The Journal of Urology*.

[5] Tsujihata M, Nonomura N, Tsujimura A, Yoshimura K, Miyagawa Y, Okuyama A (2006). Laparoscopic nephroureterectomy for upper tract transitional cell carcinoma: comparison of laparoscopic and open surgery. *European Urology*.

[6] McDonald HP, Upchurch WE, Sturdevant CE (1952). Nephro-ureterectomy: a new technique. *The Journal of Urology*.

[7] Matin SF, Gill IS (2005). Recurrence and survival following laparoscopic radical nephroureterectomy with various forms of bladder cuff control. *The Journal of Urology*.

[8] Kurzer E, Leveillee RJ, Bird VG (2006). Combining hand assisted laparoscopic nephroureterectomy with cystoscopic circumferential excision of the distal ureter without primary closure of the bladder cuff—is it safe?. *The Journal of Urology*.

[9] Vardi IY, Stern JA, Gonzalez CM, Kimm SY, Nadler RB (2006). Novel technique for management of distal ureter and en block resection of bladder cuff during hand-assisted laparoscopic nephroureterectomy. *Urology*.

[10] Nanigian DK, Smith W, Ellison LM (2006). Robot-assisted laparoscopic nephroureterectomy. *Journal of Endourology*.

[11] Agarwal DK, Khaira HS, Clarke D, Tong R (2008). Modified transurethral technique for the management of distal
ureter during laparoscopic assisted nephroureterectomy. *Urology*.

[12] El Fettouh HA, Rassweiler JJ, Schulze M (2002). Laparoscopic radical nephroureterectomy: results of an international multicenter study. *European Urology*.

[13] Muntener M, Nielsen ME, Romero FR (2007). Long-term oncologic outcome after laparoscopic radical
nephroureterectomy for upper tract transitional cell carcinoma. *European Urology*.

[14] Bariol SV, Stewart GD, McNeill SA, Tolley DA (2004). Oncological control following laparoscopic nephroureterectomy: 7-year outcome. *The Journal of Urology*.

[15] Rouprêt M, Hupertan V, Sanderson KM (2007). Oncologic control after open or laparoscopic nephroureterectomy
for upper urinary tract transitional cell carcinoma: a single
center experience. *Urology*.

[16] Manabe D, Saika T, Ebara S (2007). Comparative study of oncologic outcome of laparoscopic
nephroureterectomy and standard nephroureterectomy for upper
urinary tract transitional cell carcinoma. *Urology*.

[17] Charbit L, Gendreau M-C, Mee S, Cukier J (1991). Tumors of the upper urinary tract: 10 years of experience. *The Journal of Urology*.

[18] Hall MC, Womack S, Sagalowsky AI, Carmody T, Erickstad MD, Roehrborn CG (1998). Prognostic factors, recurrence, and survival in transitional cell carcinoma of the upper urinary tract: a 30-year experience in 252 patients. *Urology*.

[19] Ozsahin M, Zouhair A, Villà S (1999). Prognostic factors in urothelial renal pelvis and ureter tumours: a multicentre rare cancer network study. *European Journal of Cancer*.

[20] Okegawa T, Odagane A, Ide H, Horie S, Nutahara K, Higashihara E (2006). Oncological outcome of retroperitoneoscopic nephroureterectomy for upper urinary tract transitional cell carcinoma. *International Journal of Urology*.

[21] Wolf JS, Dash A, Hollenbeck BK, Johnston WK, Madii R, Montgomery JS (2005). Intermediate followup of hand assisted laparoscopic nephroureterectomy for urothelial carcinoma: factors associated with outcomes. *The Journal of Urology*.

[22] Chung S-D, Chueh S-C, Lai M-K (2007). Long-term outcome of hand-assisted laparoscopic radical nephroureterectomy for upper-tract urothelial carcinoma: comparison with open surgery. *Journal of Endourology*.

[23] Kondo T, Nakazawa H, Ito F, Hashimoto Y, Toma H, Tanabe K (2007). Impact of the extent of regional lymphadenectomy on the survival
of patients with urothelial carcinoma of the upper urinary tract. *The Journal of Urology*.

[24] Brausi MA, Gavioli M, De Luca G (2007). Retroperitoneal lymph node dissection (RPLD) in conjunction with
nephroureterectomy in the treatment of infiltrative transitional
cell carcinoma (TCC) of the upper urinary tract: impact on survival. *European Urology*.

[25] Busby J, Brown G, Matin S (2006). Efficacy of lymphadenectomy during laparoscopic radical nephroureterectomy: comparison to the open approach. *The Journal of Urology*.

